# Physical activity delays hippocampal neurodegeneration and rescues memory deficits in an Alzheimer disease mouse model

**DOI:** 10.1038/tp.2016.65

**Published:** 2016-05-03

**Authors:** M Hüttenrauch, A Brauß, A Kurdakova, H Borgers, F Klinker, D Liebetanz, G Salinas-Riester, J Wiltfang, H W Klafki, O Wirths

**Affiliations:** 1 Department of Psychiatry and Psychotherapy, University Medical Center (UMG), Georg-August-University, Göttingen, Germany; 2 Department of Clinical Neurophysiology, University Medical Center (UMG), Georg-August-University, Göttingen, Germany; 3 Department of Developmental Biochemistry, DNA Microarray and Deep-Sequencing Facility, University Medical Center (UMG), Georg-August-University, Göttingen, Germany; 4 German Center for Neurodegenerative Diseases (DZNE), Göttingen, Germany

## Abstract

The evidence for a protective role of physical activity on the risk and progression of Alzheimer's disease (AD) has been growing in the last years. Here we studied the influence of a prolonged physical and cognitive stimulation on neurodegeneration, with special emphasis on hippocampal neuron loss and associated behavioral impairment in the Tg4-42 mouse model of AD. Tg4-42 mice overexpress Aβ4-42 without any mutations, and develop an age-dependent hippocampal neuron loss associated with a severe memory decline. We demonstrate that long-term voluntary exercise diminishes CA1 neuron loss and completely rescues spatial memory deficits in different experimental settings. This was accompanied by changes in the gene expression profile of Tg4-42 mice. Deep sequencing analysis revealed an upregulation of chaperones involved in endoplasmatic reticulum protein processing, which might be intimately linked to the beneficial effects seen upon long-term exercise. We believe that we provide evidence for the first time that enhanced physical activity counteracts neuron loss and behavioral deficits in a transgenic AD mouse model. The present findings underscore the relevance of increased physical activity as a potential strategy in the prevention of dementia.

## Introduction

The probability of developing Alzheimer's disease (AD) is influenced by several factors, including diabetes mellitus, midlife obesity, midlife hypertension or physical inactivity^1^ and modern lifestyle might have a share in that risk.^2^ Analysis of population-based data revealed that about a third of AD cases worldwide might be attributable to such potentially modifiable risk factors.^3^ Several epidemiological studies suggested that physical activity results in a significantly reduced risk of dementia.^4, 5^ It has been further hypothesized that a lack of physical activity accounts for about 13% of all AD cases, leading to the prediction that a 25% increase in physical activity could potentially prevent almost 1 million cases worldwide.^6^ Friedland *et al.*^7^ have shown that people who actively engaged in mentally and physically demanding activities (such as walking, reading, gardening or jogging) during young and middle adulthood had a four times reduced probability of getting AD compared with control subjects not engaging in such activities. A reduced risk of dementia and AD was demonstrated in individuals performing leisure-time physical activity at midlife for at least twice a week, when compared with control subjects without appropriate exercise.^8^ Furthermore, a very recent study proposed an extended window of opportunity for preventive physical activity from midlife to older ages.^9^ Taken together, there is ample evidence that physical exercise reduces the risk of cognitive decline, also reflected by recent meta-studies.^10, 11, 12^ Beneficial effects were also reported for individuals already suffering from mild cognitive impairment and dementia.^13, 14^ Most of the epidemiological studies rely on retrospective assessment of activity profiles during midlife with self-reported exercise frequencies. To overcome this bias, experimental approaches applying voluntary exercise paradigms in AD mouse models in preclinical settings open the possibility of a prospective analysis with better reproducibility and comparability. The environmental enrichment paradigm is regarded as a useful tool to create a physical and intellectual stimulation for laboratory rodents and has been previously carried out in several AD mouse models^15^ with amyloid pathology such as 3xTg,^16, 17^ APP/PS1delEx9 (ref. 18) and others.

One of the major hallmarks of AD progression is severe hippocampal atrophy caused by neuron death. However by now, only a small number of AD mouse models display a reliable hippocampal neuron loss.^19^ Therefore, hardly any research investigations have been performed to test the effect of physical activity on hippocampal neuron numbers in animal models using detailed, design-based quantitative techniques. The aim of the current study was to investigate whether a prolonged physical and cognitive stimulation has a positive impact on the pathological alterations in the newly developed Tg4-42 mouse model of AD, with special emphasize on hippocampal neurodegeneration and associated behavioral impairment. This transgenic mouse model overexpresses Aβ4-42, one of the most abundant Aβ species found in human AD brain,^20^ without any mutations related to familial AD and without human amyloid precursor protein (APP) overexpression. Intracellular Aβ accumulation is most abundant in the CA1 region of the hippocampus, also present in other brain regions such as striatum and piriform cortex, and is accompanied by astro- and microgliosis. Tg4-42 mice develop an age-dependent neuron loss in the CA1 region of the hippocampus, which becomes apparent at 12 months of age in heterozygous^21^ and 6 months of age in homozygous animals,^22^ together with robust deficits in spatial reference memory in the absence of overt extracellular plaque formation.

We demonstrate here that long-term voluntary exercise diminishes CA1 neuron loss and rescues memory deficits in different experimental settings. Deep sequencing analysis suggests that upregulation of chaperones involved in endoplasmatic reticulum (ER) protein processing might be intimately linked to these beneficial effects.

## Materials and Methods

### Transgenic mice

The generation of Tg4-42 mice has been described previously.^21^ In brief, Tg4-42 mice express the human Aβ4-42 sequence combined with the signal peptide sequence of the thyrotropin-releasing hormone, ensuring secretion through the secretory pathway, under the control of the neuron-specific Thy1 promoter. Tg4-42 mice were generated and maintained on a C57Bl/6 J genetic background.^21^ All animals were handled according to the German guidelines for animal care and all experiments have been approved by the local animal care and use committee (Landesamt für Verbraucherschutz und Lebensmittelsicherheit (LAVES), Lower Saxony). Only female mice were used in the current study.

### Housing conditions

**Figure 1:**
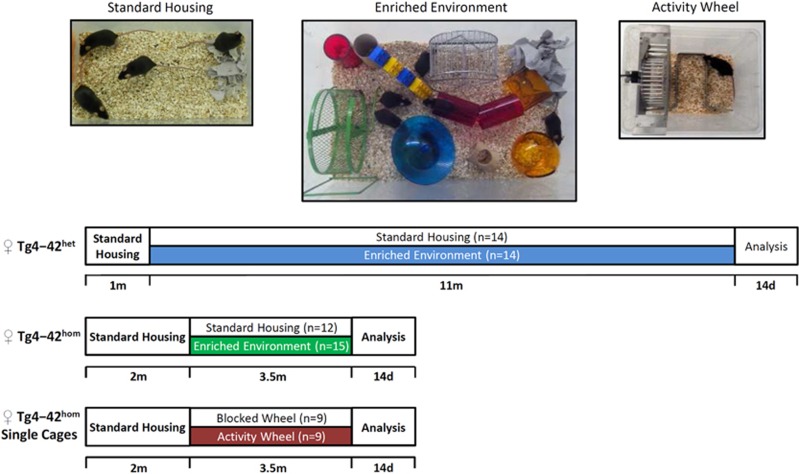
Housing paradigms. Exemplary picture of standard housing (SH), enriched environment (EE) and activity wheel (AW) cages used for the experiments, and schematic drawing of the experimental design. One-month-old Tg4-42^het^ mice were exposed to SH or EE conditions for 11 months. At 12 months mice underwent a battery of behavioral tests followed by body weight assessment, killing and tissue collection. Two-month-old Tg4-42^hom^ mice were subjected to either SH, EE or AW conditions (with blocked and free wheels) for 3.5 months, followed by behavioral testing and tissue collection.

At 1 month of age, heterozygous Tg4-42 (Tg4-42^het^) mice were randomly distributed to either standard housing (SH) or enriched environment (EE) housing conditions with 4–5 mice per cage until the age of 12 months (*n*=14 per group). Homozygous Tg4-42 (Tg4-42^hom^) mice were assigned to either of the conditions at 2 months for a duration of 3.5 months (*n*=12–15 per group). For SH, standard laboratory cages were used (33 cm × 18 cm × 14 cm), whereas for the EE living conditions, large rat cages were used (55 cm × 34 cm × 20 cm). EE cages were equipped with three running wheels, nesting material, tunnels, houses and toys, therefore offering both physical and cognitive stimulation. The entire setup was changed and rearranged weekly to increase the sense of novelty. Behavioral testing was performed for all animals housed under SH or EE conditions. For running wheel recording experiments, 2-month-old Tg4-42^hom^ mice were assigned to individual cages (22 cm × 16 cm × 14 cm) equipped with a free or blocked running wheel (*n*=9 per group) until the age of 6 months (Figure 1). Food and water were provided *ad libitum* in all conditions.

### Behavioral tasks

#### Balance beam

The balance beam task was used to assess balance and general motor function. A 1-cm dowel beam is attached to two support columns 44 cm above a padded surface. At either end of the 50-cm long beam a 9 cm × 15 cm escape platform is attached. The mouse is placed on the center of the beam and released. Each animal is given three trials during a single day of testing. The time the animal remained on the beam is recorded and the resulting time on the beam of all three trials is averaged. If an animal remains on the beam for whole 60-s trial or escapes to one of the platforms, the maximum time of 60 s is recorded.^23^

#### String suspension

The string suspension test assesses motor coordination and was described in detail previously.^23^ In brief, mice are permitted to grasp an elevated string with their forepaws and are released. During a 60-s single trial, the animals are rated with a score from 0 to 5 to assess task performance: 0=unable to remain on the string; 1=hangs only by fore- or hind paws; 2=as for 1, but with attempt to climb onto string; 3=sits on string and holds balance; 4=four paws and tail around string with lateral movement; and 5=escape to one of the platforms.

#### Accelerating rotarod

Motor performance and motor learning were tested using the rotarod (TSE Systems, Bad Homburg, Germany). Testing consists of four trials per day for 2 consecutive days with intertrial intervals of 2–3 min. Each mouse was placed on the rod, which accelerated from 1 to 45 r.p.m. over the trial time of 300 s. Trials were terminated when animals fell off (or the maximum time was reached), and latency to descent (in seconds) served as an indicator of motor coordination.

#### Morris water maze and novel object recognition tasks

**Figure 2:**
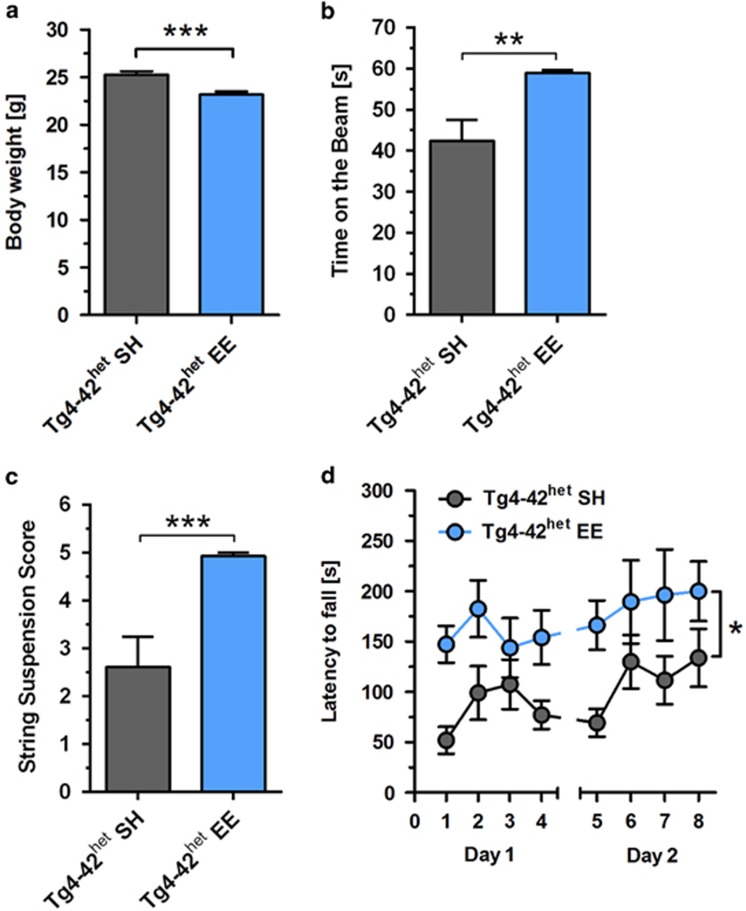
Enriched environment (EE) housing and physical activity ameliorates motor skills of Tg4-42^het^ mice. (**a**) Tg4-42^het^ EE mice showed a significantly reduced body weight compared with standard housing (SH) mice. Balance beam, string suspension and rotarod task were performed to analyze motor performance of Tg4-42^het^ SH and EE mice. Housing under enriched conditions induced an increased performance in (**b**) balance beam, (**c**) string suspension and (**d**) rotarod test. All data were given as means±s.e.m. ***P<0.001; **P<0.01; *P<0.05.

Spatial reference memory abilities were evaluated using the Morris water maze^24^ and the protocol has been fully described in previous studies.^21, 25^ The novel object recognition test was performed in an open-field box made of gray plastic (50 cm × 50 cm). On the first day, each mouse was given 5 min to explore the testing environment and become habituated. Twenty-four hours later, the exploration phase was performed in which the arena contained two identical objects (Figure 2g). Again 24 h later, mice were placed in the apparatus for the test trial, now with a familiar and a novel object. The time mice spent with each object was recorded. The objects were cleaned with alcohol between each mouse to remove any lingering scents.

### Quantification of neuron numbers using unbiased stereology

Mice were transcardially perfused with 4% paraformaldehyde in phosphate-buffered saline and brains were carefully dissected. Postfixation was carried out in 4% paraformaldehyde overnight. Stereological analysis of the hippocampal cell layer CA1 (bregma −1.22 to −3.80 mm) and the dentate gyrus (bregma −1.34 to −3.80 mm) using a stereology workstation (Olympus (Hamburg, Germany) BX51 with a motorized specimen stage for automatic sampling, StereoInvestigator 7 (MicroBrightField, Williston, ND, USA)) was performed as previously described^21, 26^ on cresyl-violet-stained sections.

### Deep sequencing analysis

Total RNA was prepared from Tg4-42^het^ SH and EE brain hemispheres (*n*=6 each) using Trifast reagent (Peqlab, Erlangen, Germany) according to the protocol of the supplier. A unit of 0.5 μg of total RNA was used as start material for the library preparation. The libraries were generated according to the TruSeq mRNA Sample Preparation Kits v2 Kit from Illumina (San Diego, CA, USA; Catalog No. RS-122-2002). For accurate quantitation of cDNA libraries we used a fluorometric-based system, the QuantiFluor dsDNA System from Promega (Mannheim, Germany). The size of final cDNA libraries was determined by using the Fragment Analyzer from Advanced Analytical Technologies (Heidelberg, Germany). The average of libraries was 320 bp. cDNA libraries were amplified and sequenced by using the cBot and HiSeq2000 from Illumina (SR; 1 × 50 bp; ca. 30 Mio reads per sample). Sequence images were transformed with Illumina software BaseCaller to bcl files, which were demultiplexed to fastq files with CASAVA v1.8.2. Quality check was done via fastqc.

Read alignment was performed using STAR v2.3.0 (code.google.com/p/rna-star) to the hg19 reference genome. Data were converted and sorted by samtools 0.1.19 (www.htslib.org) and reads per gene were counted via htseq version 0.6.1 (www-huber.embl.de/HTSeq). Data analysis was performed using R/Bioconductor (3.0.2/2.12; www.bioconductor.org) with DESeq2 and gplots packages. Candidate genes were filtered to a minimum of false discovery rate-corrected *P*-value <0.05. For functional analysis, gene ontology enrichment was tested via R-package goseq. Protein–protein interactions of differentially expressed genes were assessed using the Search Tool for the Retrieval of Interacting Genes/Proteins database^27^ (STRING v10; string.embl.de).^28^

### Reverse transcription-PCRs

Real-time reverse transcription-polymerase chain reaction was used to confirm deep sequencing results for a subset of randomly selected genes from those fulfilling the criteria of significant expression differences (*P*<0.05). Deep-frozen brain hemispheres from Tg4-42^het^ SH and EE (*n*=6 each) were homogenized in TriFast reagent (Peqlab) essentially as previously described.^29^ Primer sequences are described in the Supplementary Information, and relative expression levels were calculated using the 2^−ΔΔCt^ method^30^ and normalized to housekeeping gene β-actin. The expression ratio results of the studied transcripts are tested for significance by unpaired *t*-tests. Levels of significance were labeled as follows: ***P<0.001; **P<0.01; *P<0.05.

### Immunohistochemistry on paraffin and frozen sections

Mice were transcardially perfused with 4% paraformaldehyde in phosphate-buffered saline and brains were carefully dissected. Postfixation was carried out in 4% buffered formalin at 4 °C before paraffin embedding. Immunohistochemistry on 4 μm sagittal paraffin and frozen sections was performed as described previously.^29, 31^ The following antibodies were used: 4G8 (Aβ17-14, 1:1,0000, Covance, Princeton, NJ, USA); and doublecortin (DCX; 1:200, Santa Cruz, Dallas, TX, USA).

### Electrochemiluminescence Aβ assay

For determination of Aβ levels in whole brain hemispheres, an electrochemiluminescence total Aβ assay based on the Human (6E10) Aβ40 Ultra-Sensitive kit obtained from Meso Scale Discovery (Gaithersburg, MD, USA) was used.^32^ Here the Aβ40 detection antibody is replaced by anti-Aβ 4G8 monoclonal antibody. Therefore, the total Aβ assay uses monoclonal antibody 6E10 (directed against an aminoterminal epitope of Aβ) for capture and the monoclonal antibody 4G8 (directed against Aβ17-26) for detection. The detailed protocol is described in the Supplementary Information.

### Statistical analysis

Differences between groups were tested either two-way analysis of variance, one-way analysis of variance or unpaired *t*-tests. All data were given as mean±s.e.m. Significance levels were given as follows: ***P<0.001; **P<0.01; *P<0.05. All calculations were performed using GraphPad Prism version 6.07 for Windows (GraphPad Software, San Diego, CA, USA).

## Results

### Environmental enrichment improves motor skills of Tg4-42^het^ mice

At 1 month of age, Tg4-42^het^ mice were randomly assigned to either SH or EE conditions for 11 months (Figure 1). Mice housed under EE conditions showed a significantly reduced body weight compared with Tg4-42^het^ SH mice (*P*<0.001, Figure 2a). To analyze the effect of EE on the motor performance of Tg4-42^het^ mice, animals underwent different motor tasks. Tg4-42^het^ EE mice performed significantly better than Tg4-42^het^ SH mice in the balance beam (*P*<0.01, Figure 2b), as well as string suspension task (*P*<0.001, Figure 2c) and nearly always reached the highest score possible. During the rotarod task, the typical phases of motor skill learning, as well as motor coordination and balance were assessed. Over both days, Tg4-42^het^ SH and EE mice improved their ability to stay on the rotarod over each trial, with EE mice showing a significantly better performance compared with SH mice, demonstrated by overall higher latencies to fall (*P*<0.05, Figure 2d). The open-field test was performed in order to assess general locomotor activity and anxiety levels in Tg4-42^het^ SH and EE mice. Tg4-42^het^ mice subjected to EE conditions spent significantly more time in the center of the open-field arena compared with controls housed under standard conditions (*P*<0.01), indicating increased exploratory behavior, while the total time active did not differ between the two groups (Supplementary Figure S1).

### Enriched environment ameliorates cognitive impairment in Tg4-42^het^ and Tg4-42^hom^ mice

**Figure 3:**
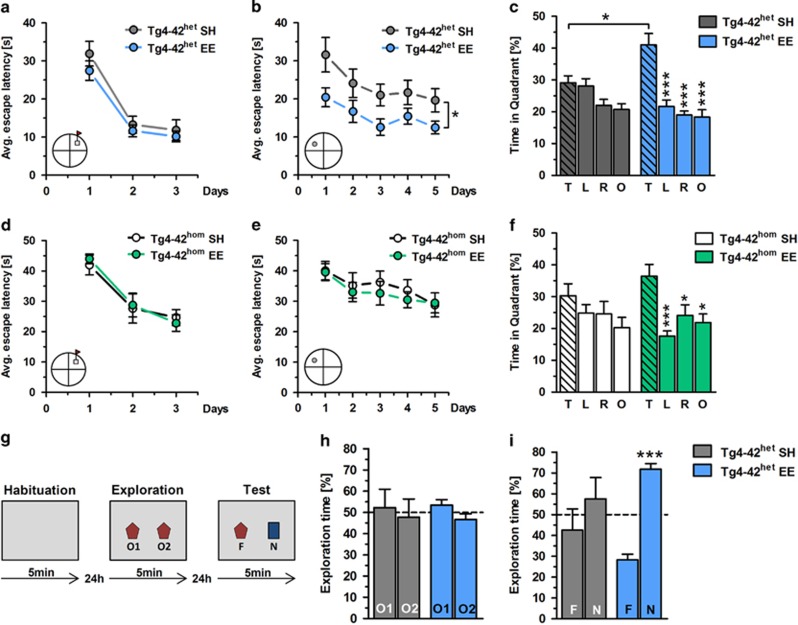
Enriched environment (EE) housing corrects cognitive deficits in Tg4-42^het^ mice and Tg4-42^hom^ mice. Tg4-42^het^ mice (12 months) and Tg4-42^hom^ mice (6 months) housed under SH or EE conditions were tested in the Morris water maze task (**a**–**f**). (**a**, **d**) All tested mice showed decreased average (Avg) escape latencies over 3 days of training, whereas no differences could be assessed between SH and EE mice. (**b**, **e**) During the acquisition training mice showed progressively reduced escape latencies over 5 days of training. Tg4-42^het^ SH mice showed an impaired spatial learning as seen by higher escape latencies over the whole training period. No differences in escape latencies could be assessed between Tg4-42^hom^ SH and EE mice (**c**, **f**) While Tg4-42^het^ SH and Tg4-42^hom^ SH mice had no preference for any of the quadrants in the probe trial, EE mice showed an intact spatial reference memory as they spent significantly more time in the target quadrant (T) compared with all the other quadrants (L, left; R, right; O, opposite) in both heterozygous and homozygous Tg4-42 mice. Recognition memory was tested using the novel object recognition task (NOR) (**g**–**i**). (**g**) Schematic representation of the NOR task. (**h**) In the exploration phase, SH and EE mice spent ~50% of the time with the novel (N) and familiar (F) object. (**i**) During the test trial, EE mice showed a clear preference for the novel object, whereas SH mice showed no preference for any of the objects. All data were given as means±s.e.m. ***P<0.001; *P<0.05.

Severe memory deficits have been observed in 12-month-old Tg4-42^het^ and 6-month-old Tg4-42^hom^ mice. Spatial reference memory of SH- and EE-housed heterozygous and homozygous Tg4-42 mice was assessed using the Morris water maze. Both Tg4-42^het^ and Tg4-42^hom^ SH/EE mice showed strongly decreased escape latencies over 3 days of cued training (Figures 2a and d). While Tg4-42^het^ SH/EE mice showed comparable swimming speeds during the cued training, Tg4-42^hom^ EE mice swam faster compared with Tg4-42^hom^ SH mice (*P*<0.001; Supplementary Figure S2). During the acquisition training phase, Tg4-42^het^ SH mice showed significantly longer escape latencies over the whole training period compared with Tg4-42^het^ EE mice (*P*<0.05, Figure 3b), whereas no such differences were observed in Tg4-42^hom^ mice (Figure 3e). No differences in swimming speed were noted during the acquisition training period in Tg4-42^het^ SH/EE mice, whereas Tg4-42^hom^ EE mice were faster compared with Tg4-42^hom^ SH mice (*P*<0.05; Supplementary Figure S2). In the probe trial, neither Tg4-42^het^ SH nor Tg4-42^hom^ SH mice showed a preference for the target quadrant. In contrast, mice maintained under enriched conditions showed a preservation of spatial reference memory in both genotypes as they spent significantly more time in the target quadrant (T) than in the left (L), right (R) or opposite (O) quadrants (Figures 3c and f). The swimming speed of Tg4-42^het^ EE mice was slightly increased compared with SH mice in the probe trial (*P*<0.05), whereas no differences were observed in Tg4-42^hom^ SH/EE mice during the probe trial test (Supplementary Figure S2). A control group of singly housed Tg4-42^hom^ mice in running wheel-equipped cages could not be tested by the Morris water maze paradigm due to an unexpected hyperflexion of the tail (Supplementary Figure S3).

### Improved recognition memory performance in Tg4-42^het^ EE mice

Recognition memory was tested using the novel object recognition task (Figure 3g). On the exploration day, Tg4-42^het^ SH and EE mice explored two identical objects for equal durations (Figure 3h). When tested for recognition memory 24 h later, EE mice spent significantly more time with the novel object compared with the familiar object (*P*<0.001), whereas Tg4-42^het^ SH mice did not show a preference for any of the objects (Figure 3i).

To assess a potential housing effect on hippocampus-related spatial working memory, Tg4-42^het^ SH and EE mice were tested in the Cross Maze task. Tg4-42^het^ SH mice did not show an impaired spatial working memory as they performed better than chance level (indicated by the dotted line). However, Tg4-42^het^ EE mice showed a significantly improved performance evident by increased alternation rates compared with SH mice (*P*<0.05). No differences in total arm entries could be detected between the two groups and therefore the enhanced alternation rate of Tg4-42^het^ EE mice cannot be attributed to altered explorative behavior (Supplementary Figure S1).

### Voluntary exercise decreases neuron loss in Tg4-42^het^ and Tg4-42^hom^ mice

**Figure 4:**
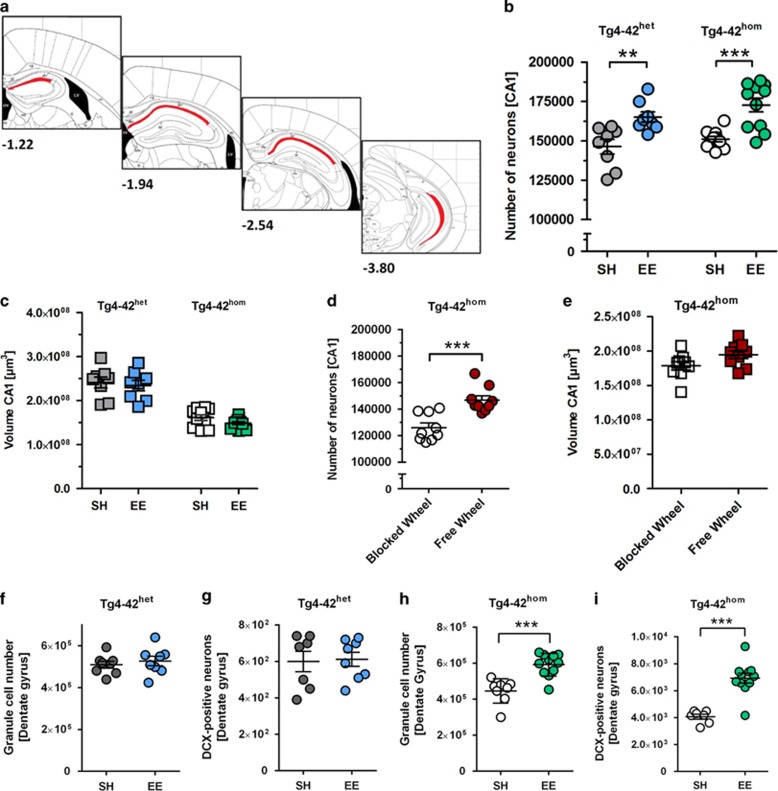
Exercise decreases the hippocampal neuronal loss in the CA1 area of Tg4-42^het^ and Tg4-42^hom^ mice and increases neurogenesis in Tg4-42^hom^ mice. (**a**) Schematic drawing of the counting area (modified from Paxinos and Franklin^34^). (**b**) Design-based stereology was performed to determine the number of CA1 pyramidal cells in 12-month Tg4-42^het^ and 6-month Tg4-42^hom^ mice. Significantly increased neuron numbers could be detected in enriched environment (EE) Tg4-42^het^ and Tg4-42^hom^ mice (+12.8% and +14.4%, respectively) compared with standard housing (SH) littermates. (**c**) No CA1 volume differences could be detected between SH and EE Tg4-42^het^ and Tg4-42^hom^ mice. (**d**) Tg4-42^hom^ mice with access to a free wheel showed a 16.5% increased CA1 neuron number compared with animals with blocked wheel cages, but (**e**) no volume difference. (**f**, **h**) Quantification of dentate gyrus (DG) granule cells revealed no differences between SH and EE Tg4-42^het^ mice, while EE Tg4-42^hom^ mice displayed significantly higher granule cell numbers in the DG compared with SH controls. (**g**, **i**) Quantification of doublecortin (DCX)-positive neurons in the subgranular zone of the DG revealed no increased neurogenesis in 12-month Tg4-42^het^ mice upon EE housing. In contrast, 6-month Tg4-42^hom^ EE mice showed a 32.6% increase of subgranular DCX-positive neurons compared with Tg4-42^hom^ SH mice. All data were given as means±s.e.m. ***P<0.001; **P<0.01.

Hemizygous 12-month-old Tg4-42 mice show a 49% neuron loss in the CA1 region of the hippocampus compared with WT mice.^21^ Homozygous 6-month-old Tg4-42 mice also display a CA1 neuron loss of 50%. To analyze if a prolonged cognitive and physical stimulation positively impacts on CA1 neuron numbers of Tg4-42 mice, unbiased design-based stereological analyses were performed. Tg4-42^het^ mice maintained under enriched conditions showed a 12.8% higher number of CA1 pyramidal cells compared with Tg4-42^het^ SH mice (*P*<0.01). In good agreement, Tg4-42^hom^ EE mice displayed a 14.3% increased CA1 neuron number compared with SH littermates (*P*<0.001, Figure 4b). Analysis of the CA1 volume did not show any differences related to housing conditions in Tg4-42^het^ or Tg4-42^hom^ mice (Figure 4c). As group housing did not allow predictions about running wheel use of individual animals and in order to assess whether physical activity alone exerts beneficial effects, singly housed Tg4-42^hom^ mice in cages with either free or blocked running wheels for 3.5 months were analyzed as an additional control group. Mice with access to free wheels showed an increased weekly running distance that corresponded to ~30 km at the end of the trial (Supplementary Figure S3). Single housed Tg4-42^hom^ mice subjected to free wheel conditions revealed a 16.5% increase in CA1 neuron numbers compared with the blocked wheel control group (*P*<0.001, Figure 4d), but no significant differences in CA1 volume (Figure 4e). Stereological analysis of dentate gyrus granule cells revealed unchanged neuron numbers in 12-month-old Tg4-42^het^ mice (Figure 4f), as well as an unchanged number of DCX-positive neurons indicating no changes in neurogenesis (Figure 4g). In contrast, 6-month-old EE-housed Tg4-42^hom^ mice showed a significantly increased number of dentate gyrus granule cells (+32.6%) compared with SH littermates (*P*<0.001; Figure 4h), also reflected in a significantly elevated neurogenesis rate (*P*<0.001; Figure 4i) and a more ramified shape of DCX-positive neurons in Tg4-42^hom^ EE mice (Supplementary Figure S4). According to the categorization of dendritic morphology of DCX-positive cells from Plümpe *et al.*, cells of sedentary Tg4-42^hom^ mice belong to categories A–D while DCX-positive cells of enriched mice belong to categories E–F. Cells of category A and B have no or very short processes, while C and D cells show medium processes and E and F cells display a strong dendritic branching.^33^

In order to evaluate whether the increased number of CA1 pyramidal neurons in mice kept under enriched conditions was accompanied by decreased levels of Aβ4-42, immunohistochemical stainings of Aβ were performed in Tg4-42^het^ SH and EE mice. Qualitatively, no differences in Aβ immunoreactivity could be seen between standard and enriched housed mice (Supplementary Figure S5). To further confirm this result quantitatively, Aβ levels of Tg4-42^het^ SH and EE mice were measured using an electrochemiluminescence Aβ assay. No significant differences in Aβ levels could be detected in both groups (Supplementary Figure S5).

### Housing under enriched conditions changes the gene expression profile of Tg4-42^het^ mice

**Figure 5:**
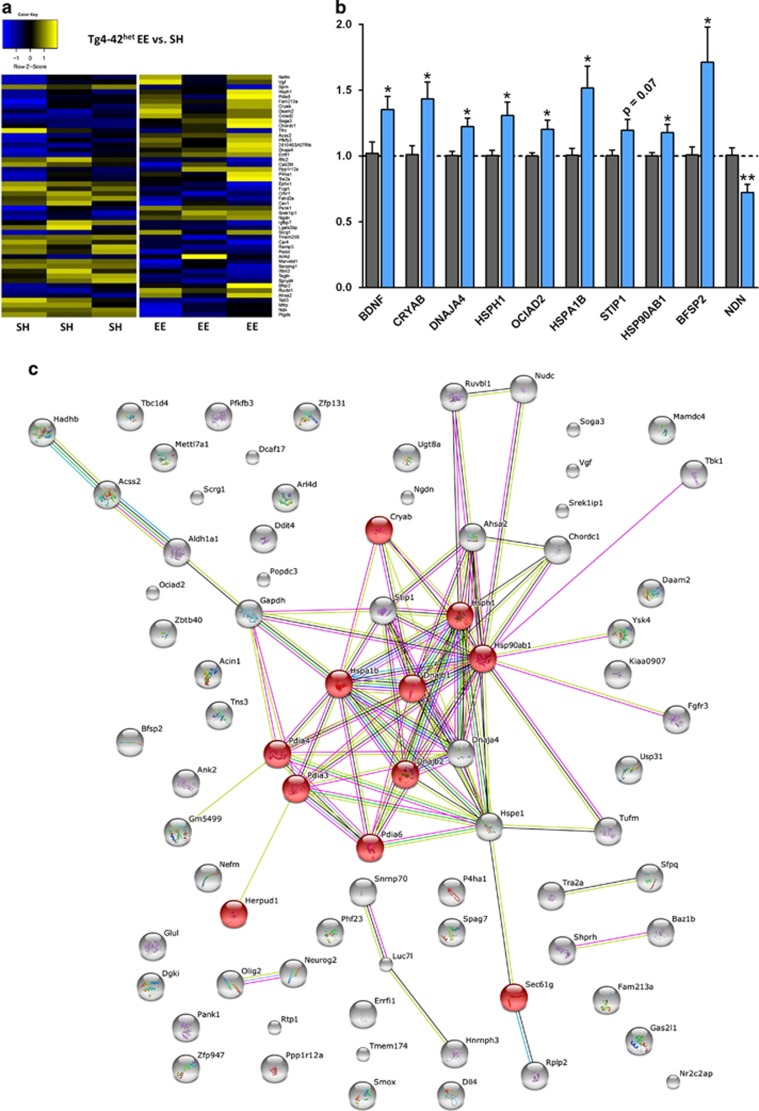
Lifelong exercise changes the gene expression profile of Tg4-42^het^ mice. (**a**) Heatmap of differentially expressed genes (DEGs) between standard housing (SH) and enriched environment (EE) Tg4-42^het^ mice. Each column represents a pooled sample of two brain hemispheres and each row represents one of the top 60 genes that were differentially expressed. Yellow indicates upregulated and blue indicates downregulated gene expression. (**b**) Reverse transcription-PCR validation of deep sequencing results normalized to β-actin. (**c**) A protein-protein interaction network of genes found to be upregulated upon EE was created using STRING10 and showed enrichment of chaperones involved in endoplasmic reticulum protein processing. All data were given as means±s.e.m. *P<0.05, **P<0.01.

To analyze whether long-term effects of an EE and voluntary exercise paradigm influence the gene expression profile of Tg4-42^het^ brains, deep sequencing analysis on whole brain hemispheres were performed. Prolonged exposure to enriched conditions changed the expression of 155 genes statistically significant (Supplementary Table 2). In all, 80 genes were upregulated and 75 genes were downregulated in Tg4-42^het^ EE mice compared with SH littermate controls (Figure 5a and Supplementary Figure S6). To validate the differentially expressed genes identified by deep sequencing analysis, both up- and downregulated candidate genes were randomly selected and verified by quantitative reverse transcription-PCR. For all selected genes, the quantitative reverse transcription-PCR analysis confirmed the expression levels of the deep sequencing results (Figure 5b). Some candidate genes chosen at random were also analyzed in Tg4-42^het^ compared with age-matched WT mice, showing that they were not regulated in the absence of an EE paradigm (Supplementary Figure S7). A gene ontology analysis of the upregulated genes using the String 10 software package revealed an association with the following gene ontology terms: biological processes: ‘protein folding (*P*=7.750E-6), ‘response to stress' (*P*=5.689E-3) and ‘negative regulation of inclusion body assembly' (*P*=1.830E-2). A Kyoto Encyclopedia of genes and genomes (KEGG) pathway analysis revealed a significant association with the pathway ‘protein processing in endoplasmic reticulum' (*P*=3.150E-9). The involved genes are indicated in red (Figure 5c).

## Discussion

Our group recently created a novel AD mouse model that exclusively expresses N-truncated Aβ4-42. The expression of Aβ4-42 induces an age-dependent CA1 neuron loss associated with a severe memory decline.^21^ The aim of the present study was to elucidate whether a prolonged physical and/or cognitive stimulation can counteract CA1 neuron loss and ameliorate behavioral deficits in Tg4-42 mice. Therefore, the EE paradigm, offering both physical and cognitive stimulation, was started before disease onset and continued until 6 or 12 months of age in homo- and hemizygous mice, respectively, terminating at time points were SH mice display robust behavioral deficits and profound loss of CA1 hippocampal neurons.

Animals allowed to exercise revealed a significantly reduced body weight compared with standard housed littermates, which is in agreement with an increased body fitness due to long-term running, confirming observations in previous studies using related paradigms.^35^ Upon enriched housing conditions, an improved performance in balance beam, string suspension and the rotarod test was detected. These observations are in accordance with recent studies in AD patients showing beneficial effects of exercise on their physical performance and mobility.^36^ As a further indicator for the effectiveness of our paradigm, increased expression levels of the brain-derived neurotrophic factor (BDNF) were measured, which has been previously described to be induced following voluntary exercise in rodents,^37, 38^ as well as in AD patients.^39^

Robust deficits in spatial reference memory have been previously reported in heterozygous 12-month-old Tg4-42 mice.^21^ A comparable phenotype indicating severe hippocampal functional disturbance is present in 6-month-old homozygous Tg4-42 mice.^22^ Long-term voluntary exercise and cognitive stimulation provided by a combined EE paradigm completely prevented this phenotype as shown by a rescued performance in the Morris water maze task. This finding is in line with other studies showing an improved memory task performance upon long-term running in rodents,^40, 41, 42^ and further supports findings from recent clinical data showing that aerobic fitness improves the memory performance of healthy individuals and mild cognitive impairment patients.^14, 43, 44^ The novel object recognition task is another hippocampal-dependent memory test assessing non-spatial learning and memory.^45, 46^ We demonstrate for the first time that 12-month-old Tg4-42^het^ mice show impaired recognition memory, which can be completely rescued using EE housing conditions.

Most interestingly, improvement in cognition of both hemi- and homozygous Tg4-42 mice is accompanied by a significantly diminished CA1 neuron loss in EE animals compared with their SH littermates. In order to dissect whether the impact on neuron loss can be attributed to our EE paradigm or voluntary exercise alone, a control experiment using singly housed mice in cages equipped with blocked or free running wheels was performed. The daily running distance of 4.2 km on average is in the range that has been previously reported for the C57Bl/6 J strain.^47^ The finding of significantly increased CA1 neuron numbers in singly housed Tg4-42^hom^ mice suggests that increased physical activity is sufficient to modify pathological events triggering neuronal cell death. Owing to an unexpected tail hyperflexion phenotype, which broke experimenter's blindness and precluded Morris water maze testing due to swimming incapability, the stereological data of this group cannot be backed-up with behavioral data. Literature on this phenomenon is scarce, but it has been reported that such bodily transformations can occur after extensive and regular running wheel usage for periods of 8 weeks.^48^ The fact that effects on CA1 neuron number are comparable between EE and singly housed Tg4-42^hom^ mice supports previously published data suggesting that running is the critical stimulus responsible for the beneficial effects seen in EE paradigms.^16, 49, 50^

To the best of our knowledge, the current report is the first quantitative study showing a beneficial effect of physical activity on CA1 neuronal loss using unbiased, design-based stereology in an AD mouse model. We previously published an EE study on APP/PS1KI mice,^51^ demonstrating that the housing condition had no impact on the selective loss of hippocampal CA1 neurons in that model.^31^ A possible explanation for these conflicting results could be that the APP/PS1KI mouse model represents a robust and aggressive model of familial AD incorporating several mutations, which cannot be counteracted efficiently by a rather mild intervention like voluntary exercise. In contrast, the Tg4-42 model only expresses Aβ4-42 without any mutations and therefore rather represents a model of the sporadic form of AD, likely to be more susceptible to modification through environmental factors.

Although Tg4-42^hom^ mice also revealed an increased number of dentate gyrus granule cells, no such difference could be detected in Tg4-42^het^ mice. This might be due to a general age-related reduction in rodent neurogenesis,^52^ reflected by the fact that 6-month-old Tg4-42^hom^ mice show a ~7-fold increased number of DCX-positive cells compared with aged Tg4-42^het^ mice (Figure 3). Our observation that DCX-positive cells display strong dendritic branching in enriched Tg4-42^hom^ mice is in good agreement with previous reports claiming that running acts as a stimulus for enhanced dendritic arborization of newborn dentate granule cells.^38, 53^

Other AD mouse models that have been subjected to EE/voluntary exercise paradigms show a decreased amyloid load.^54, 55^ Tg4-42 mice secrete Aβ4-42 that readily forms soluble, neurotoxic aggregates, predominantly in the CA1 region, without developing extracellular amyloid plaques.^21^ Improved memory performance and reduced neuron loss in Tg4-42^het^ mice upon EE was not accompanied by decreased levels of Aβ in the present study. Therefore, it can be speculated that the cognitive improvement and the neuroprotective effect induced by voluntary exercise in our experiments is not dependent on a simple reduction in Aβ4-42 levels. Similar observations have been made in other models demonstrating improved cognitive performance despite unchanged Aβ levels^56^ or amyloid plaque load.^31, 57^

To elucidate the molecular mechanisms leading to the beneficial effects in EE-housed Tg4-42^het^ mice we performed a whole-brain transcriptome analysis. One important candidate molecule is BDNF, which has been found to be upregulated in the current experiment. BDNF has been previously shown to have a critical role in the formation and consolidation of memory^58^ and has been demonstrated to ameliorate neuron loss in an AD mouse model using a gene-delivery paradigm.^59^ In addition, BDNF has a critical role in spatial reference and object–place memory, as recently demonstrated in mice with a partial BDNF knockdown.^60^ The most strongly downregulated gene is Necdin (*Ndn*), a neuronal protein implicated in Prader–Willi syndrome.^61^ Interestingly, *Ndn*-deficient mice have been shown to display improved learning and memory in the Morris water maze.^62^ Among the upregulated genes, a striking number of transcripts belong to chaperone families implicated in ER protein processing (*Cryab*, *Hspa1b*, *Hsp90ab1*, *Dnajab1*, *Dnajb2*, *Hsph1*, *Pdia3*, *Pdia4*, *Pdia6*, *Sec61g* and *Herpud1*), in particular to the ER-associated degradation pathway. These molecular chaperones have important roles during protein synthesis in assisting proper translocation, modification and folding, and some of them, such as HSP90AB1, have been demonstrated to be significantly repressed in AD.^63^ The ER-associated degradation pathway is involved in ER quality control leading to the disposal of misfolded proteins and finally degradation through the proteasome.^64^ It has been shown before that the induction of Hsps in the brain can be triggered by exercise in both humans and rodents.^65^ Their fundamental role is to protect cells under stress conditions from damage by refolding or degrading misfolded proteins and therewith maintaining the functionality of the proteome. Owing to the tendency of Aβ to misfold into toxic oligomers, the role of molecular chaperones in AD has gained particular interest. Overexpression of Aβ42 in neuronal cultures led to the rapid induction of Hsp70, and virally overexpressed Hsp70 protected from the toxic effects of intracellular Aβ accumulation.^66^ The HSP40-homolog DNAJB6 has been shown to modulate Aβ42 aggregation *in vitro* by binding to Aβ fibrils and inhibiting their elongation and growth,^67^ and the HSP40-homologs identified in the current analysis might act in a similar manner. A related function had been proposed for αB-crystallin,^68^ representing the highest upregulated gene in the current analysis, which also potently inhibited Aβ1-40 aggregation.^69^ Hsp70/40 and Hsp90 have been shown to block Aβ self-assembly at substoichometric concentrations, mainly by causing structural changes in oligomers.^70^ We have previously demonstrated the strong tendency of Aβ4-42 to form stable aggregates by nuclear magnetic resonance-spectroscopy and dynamic light-scattering analyses.^21^ Taken together, the upregulation of a chaperome subnetwork upon long-term exercise found in our study suggests an interaction of Hsps with Aβ4-42, thereby inducing conformational changes resulting in less neurotoxicity, despite unchanged total Aβ levels. In good agreement, polyglutamine-induced neurodegeneration in a drosophila model is suppressed by Hsp70 expression without an effect on the formation of nuclear inclusions.^71^

The induction of growth factors such as BDNF, as well as the upregulation of genes that encode cytoprotective proteins such as heat-shock proteins, is carried out by neurons as a consequence of mild stressors such as exercise or caloric restriction. This might represent a synergistically acting ‘pre-conditioning' phenomenon needed to increase cellular abilities to resist more severe stress,^72^ including accumulation of misfolded amyloid peptides. Therefore, a combination of the induction of ER-associated degradation pathway proteins, as well as neurotrophins such as BDNF might be responsible for the beneficial effects seen on the pathology of Tg4-42 mice.

In conclusion, we believe that our study is the first to demonstrate that long-term physical activity exerts a preventive effect on Aβ-induced neuron loss in the Tg4-42 transgenic mouse model of AD, thereby supporting epidemiological data on human AD obtained in retrospective studies. The ameliorated neurodegeneration is accompanied by an improved motor performance, complete memory recovery and induction of ER stress chaperones.

## Supplementary Material

Supplementary Informations
