# Supplementary material for: Physical activity delays hippocampal neurodegeneration and rescues memory deficits in an Alzheimer disease mouse model
Source: Transl Psychiatry. 2016 May 3;6(5):e800–. doi: 10.1038/tp.2016.65 (PMC5070068; doi:10.1038/tp.2016.65)
Supplement: Supplementary Informations [file tp201665x1.pdf]

Supplementary Information: **Hüttenrauch M. et al.**

Physical activity delays hippocampal neurodegeneration and rescues memory deficits in an Alzheimer disease mouse model

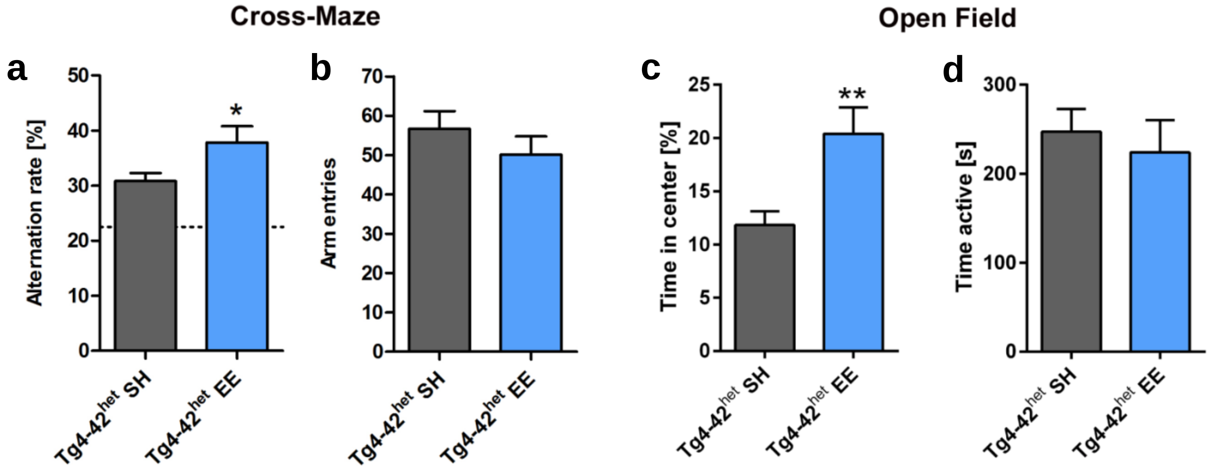

Supplementary Figure S1:

Spatial working memory was evaluated using the cross-maze task (**a**, **b**). (**a**) Tg4-42^{het} EE mice displayed significantly higher alternation rates in the cross-maze task. The dotted line represents chance level. (**b**) The enhanced alternation is not due to higher explorative behavior as there is no difference in total arm entries between SH and EE mice. (**c**) Enriched housed Tg4-42^{het} mice spent more time in the center of the open field compared with Tg4-42^{het} SH mice. (**d**) No differences in activity levels could be detected between standard and enriched housed Tg4-42^{het} mice during the open field task. All data were given as means ± standard error of the mean (SEM) (**$p < 0.01$; *$p < 0.05$).

Cross-Maze: Working memory was assessed by analyzing spontaneous alternation behavior in the Cross-Maze task. The Cross-Maze consists of 4 arms, constructed from black plastic material with arm sizes of 30 cm x 8 cm. During 10 min test sessions, each mouse was randomly placed in one arm and allowed to move freely through the maze. Alternation was defined as successive entries into the four arms in overlapping quadruple sets. An entry was defined to be successive as soon as a mouse enters an arm with all four paws. The percent alternation was calculated as the ratio of actual to possible alternations. In order to diminish odor cues, the maze was cleaned with a solution containing 30% ethanol, 60% water and 10% odorless soap (Cotel et al., Neurobiol Aging 33: 96-107 (2012)).

Open Field: The open field test was used to asses both exploratory behavior and locomotor activity. The mice were tested using an open field box made of grey plastic with 50 x 50 cm surface area and 38 cm-high walls. Monitoring was done by an automated tracking system (AnyMaze, Stoelting). The percentage of time spent in the central part (20 x 20 cm) and the total active time was recorded (Jawhar et al., Neurobiol Aging 33: 196.e29–196.e40 (2012)).

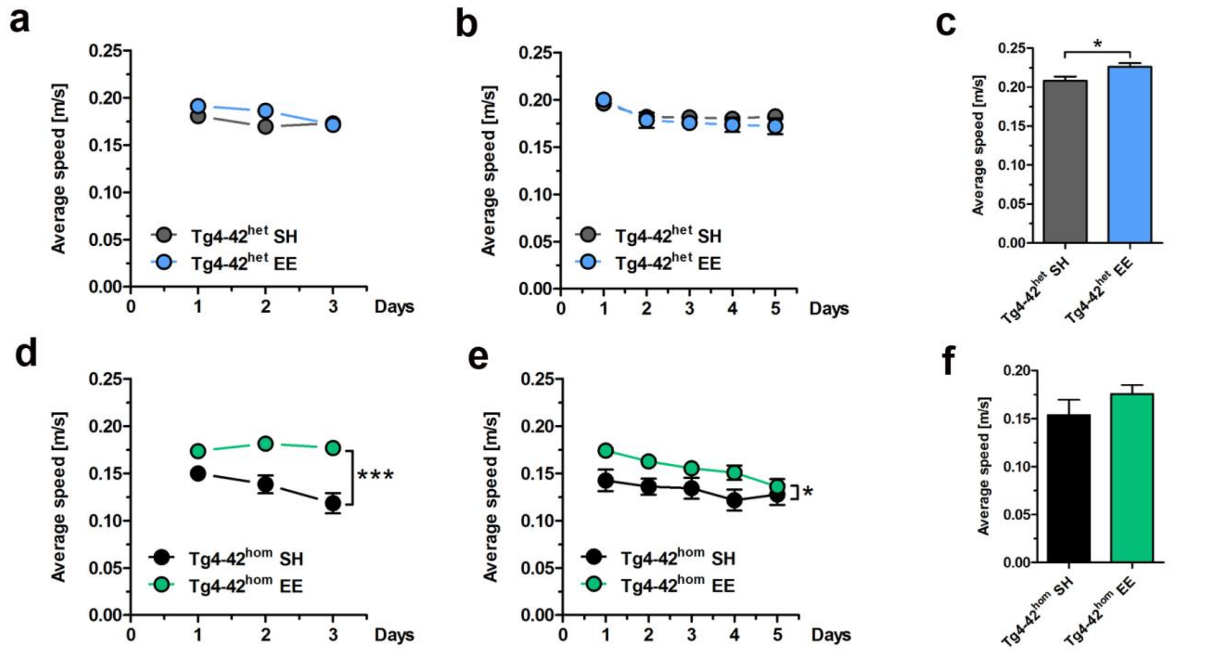

Supplementary Figure S2:
(**a**, **d**) In the cued training phase of the Morris water maze test, Tg4-42^{het} SH or EE mice did not show any differences in the swimming speed, while Tg4-42^{hom} EE mice swam significantly faster compared to SH mice. (**b**, **e**) Tg4-42^{het} SH and EE mice showed similar swimming speeds during the acquisition training trials while Tg4-42^{hom} EE mice again swam faster compared to standard housed mice. (**c**, **f**) During the probe trial, Tg4-42^{het} EE mice showed a slightly increased swimming speed compared to Tg4-42^{het} SH mice while there was no speed difference between groups in Tg4-42^{hom} mice. All data were given as means ± standard error of the mean (SEM) (****p* < 0.001; **p* < 0.05).

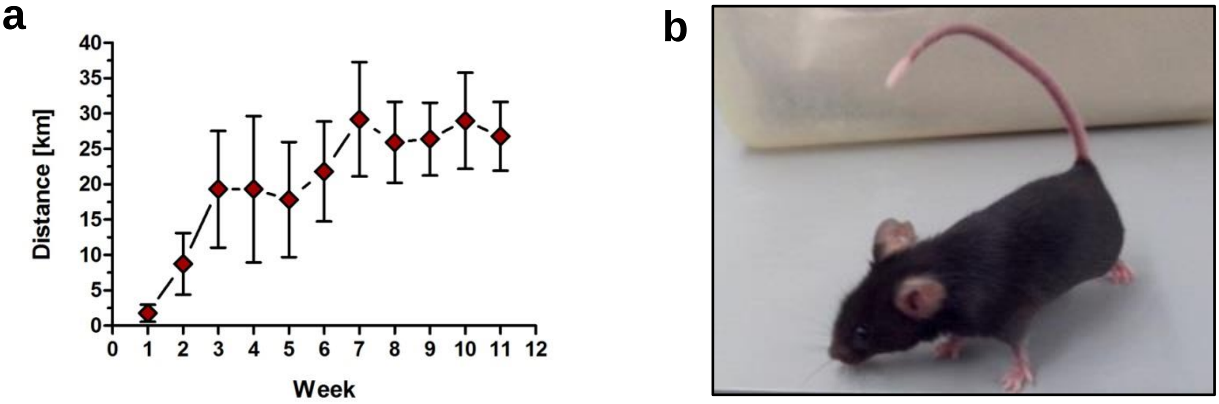

Supplementary Figure S3:

(**a**) Overview of voluntary wheel running performance of Tg4-42^{hom} mice in the recording period of 11 weeks. The average distance (km) per week is represented. (**b**) Exemplary picture of the tail hyperflexion phenotype due to the regular running wheel usage. All data were given as means ± standard error of the mean (SEM).

During the weeks of running wheel training mice were housed in single cages with food and water ad libitum. All animals had continuous voluntary access to a running wheel with a diameter of 11.3 cm placed inside the cage. A rotation sensor connected to the running wheel axis transmitted running activity with a resolution of 1/16 revolution and a sampling rate of 1/0.48 s to a customized recording device (Boenig und Kallenbach oHG, Dortmund, Germany). From this raw data, the average weekly running distance (km) was calculated and visualized using a custom-designed Matlab (The MathWorks, Inc., Natick, MA, USA) program. Animals were divided into two groups of equal size (n = 9). The running wheels of one group were freely movable whereas the running wheels of the other group were blocked, preventing the animals from running activity while providing the same enrichment of the environment.

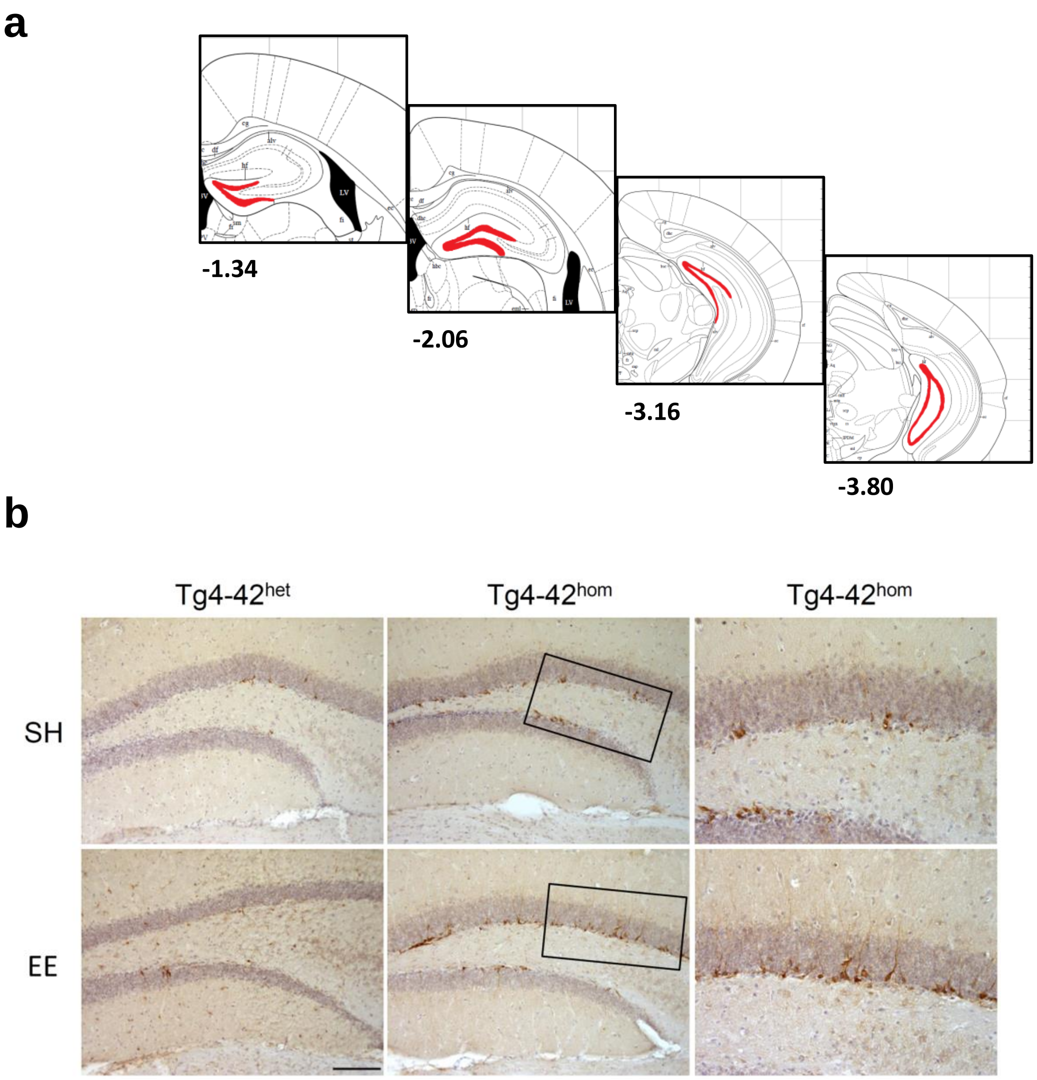

Supplementary Figure S4:
(**a**) Schematic drawing of the counting area. The dentate gyrus was quantified from Bregma -1.34 to -3.80. Figures were created using the mouse atlas by Paxinos and Franklin [57]. (**b**) New-born doublecortin (DCX)-positive neurons were stained in the subgranular zone (SGZ) of the dentate gyrus (DG). Adult neurogenesis was found to be marginal in 12-month-old Tg4-42^{het} mice which could not be restored by enriched living conditions at that age. However, 6-months-old Tg4-42^{hom} mice showed profound neurogenesis which was increased after 4 months of EE. A higher magnification shows that new-born neurons in enriched Tg4-42^{hom} mice are longer and more branched compared to standard housed controls. Scale bar: 100 µm.

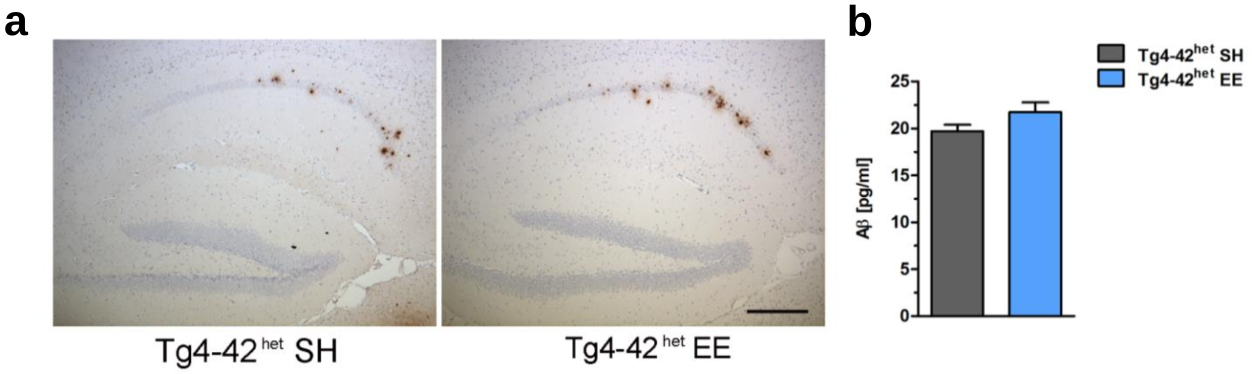

Supplementary Figure S5:

Housing condition has no impact on Aβ levels in Tg4-42^{het} mice. (**a**) Immunostaining in the hippocampus using the 4G8 antibody against Aβ (Aβ 17-14, Covance) showed a comparable staining in the CA1 region of standard and enriched housed Tg4-42^{het} mice. (**b**) Aβ levels of whole brain hemispheres were quantified using an electrochemiluminescence assay. No difference could be detected in Tg4-42^{het} SH and EE mice (n = 6 per group). Scale bar: 200 µm. Data were given as means ± standard error of the mean (SEM).

Electrochemiluminescence Assay

Whole brain hemispheres were homogenized with 10 strokes of a glas-teflon homogenizer (800 rpm, CAT) in 700 µl lysis buffer (120 mM NaCl, 50 mM Tris, 1 % Triton X-100, 1 x Complete Mini-Protease Inhibitor, 1 x Complete Mini-Phosphatase inhibitor, dissolved in 10 ml ddH$_2$O, pH 7.5) per 100 mg brain tissue. After centrifugation at 17000 x g at 4°C for 20 min, the supernatant containing the protein was stored at -80 °C until further use.
Protein concentrations of lysates were determined using the Roti®-Quant universal kit according to the instructions of the supplier (Carl Roth).
For determination of Aβ levels in whole brain hemispheres, an electrochemiluminescence total Aβ assay obtained from Meso Scale Discovery (Gaithersburg, MD, USA) was used. The Aβ assay is based on the Human (6E10) Abeta 40 Ultra-Sensitive kit. Here, the Aβ40 detection antibody is replaced by anti-Aβ 4G8 monoclonal antibody. Therefore, the total Aβ assay employs monoclonal antibody 6E10 (directed against an aminoterminal epitope of Aβ) for capture and the monoclonal antibody 4G8 (directed against Aβ17-26) for detection. As previously shown, 6E10 detects Aβ4-42 equally well compared to full-length Aβ1-42 or other N-truncated variants like Aβ2-42 or Aβ3-42 (Vanderstichele et al., Clin Chem 51(9): 1650-1660 (2005)).
The assay was performed according to the protocol of the manufacturer and readout on a MSD QuickPLex SQ 120. In brief, a 96-well plate pre-coated with an Aβ antibody (6E10) was blocked with 3% BSA under shaking conditions at room temperature for 1 h. After 3 washing steps with 150 µl/well of 1X Tris Wash Buffer, 25µl of 2mg/ml protein lysates or calibrator was added per well and incubated under shaking conditions at room temperature for 1 h. After 3 additional washing steps, 25 µl of detection antibody solution (4G8) was added and again incubated with shaking for 1 h at room temperature in the dark. Upon 3 more washing steps, 150 µl of 1X Read Buffer T was added to each well and plate was read on MSD instrument.

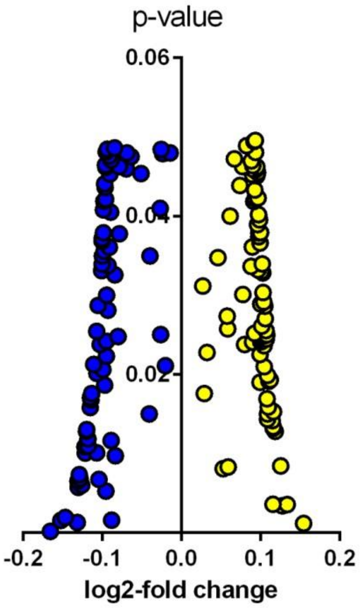

Supplementary Figure S6:
Volcano plots of the significantly differentially expressed genes showing log2-fold-change (x-axis) and p-value (y-axis) of DEGs comparing Tg4-42het SH versus EE. Significantly down-regulated genes (n =75) are depicted in dark blue and up-regulated genes (n = 80) in yellow.

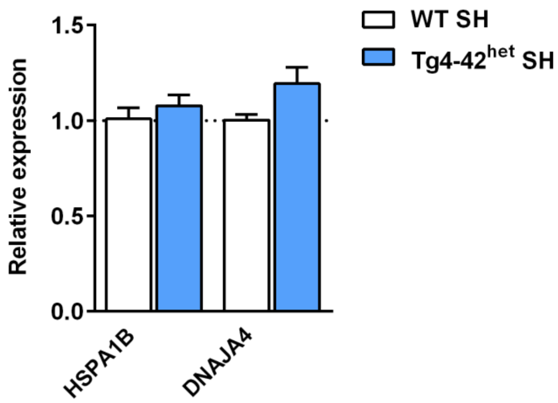

Supplementary Figure S7:
Analysis of members of the HSP70 (HSPA1B) or HSP40 (DNAJA4) family revealed that these candidate genes are not per se altered in standard housed heterozygous Tg4-42 mice compared to standard housed WT mice. No significant differences in expression levels were detected (n = 6 per group).

Supplementary Table 1: Primer sequences used in the present study

Name	Forward 5' – 3'	Reverse 3' - 5'	Amplicon size
DNAJA4	ATTGCCTGTTCTCCACCTTG	CAAGTACCACCCGGACAAGA	123
OCIAD2	GATTTGGGGCAAAACAACAG	ACGGCAATAGAAGAAAACGC	118
BFSP2	GCGTTTTCTAGGACAGCCTC	GAACTGGAAACACAACTGCG	125
HSPH1	GGCTTCTACAGGCAGCTCAA	CAGAAGAAAGCAAAACCCCA	116
NDN	GTGTGGAGATTGGTCAGCCT	AAAGAGGTCATGGGCAGCTA	110
STIP1	AGCACTGTAAGGCATCATCAA	GAATTCGATTCAACGGGGT	111
HSP90AB1	ACGGACCCTTCTAAGTTGGAC	TCAGCCTTGGTCATGCCAATG	113
CRYAB	GATCCGGTACTTCCTGTGGA	TCTCTCCGGAGGAACTCAAA	122
HSPA1B	ATGACCTCCTGGCACTTGTC	GCTCGAATCCTATGCCTTCA	111
BDNF	GCCTTCATGCAACCGAAGTA	TGAGTCTCCAGGACAGCAAA	103

Supplementary Table 2: Significantly regulated genes identified by RNA-Sequencing in Tg4-42^{het} SH vs Tg4-42^{het} EE:

ID	Gene name	Gene description	log2 Fold change	Adjusted p-value
MGI: 88516	Cryab	crystallin, alpha B	0,154	1,2E-03
MGI: 105053	Hsph1	heat shock 105kDa/110kDa protein 1	0,134	3,6E-03
MGI: 1916377	Ociad2	OCIA domain containing 2	0,127	3,4E-03
MGI:1927638	Dnaja4	DnaJ (Hsp40) homolog, subfamily A, member 4	0,125	8,4E-03
MGI:1933155	Arl4d	ADP-ribosylation factor-like 4D	0,118	1,3E-02
MGI:1933972	Tra2a	transformer 2 alpha homolog (Drosophila)	0,116	1,5E-02
MGI:1343180	Vgf	VGF nerve growth factor inducible	0,116	1,3E-02
MGI:1921405	Errfi1	ERBB receptor feedback inhibitor 1	0,116	1,3E-02
MGI:1333828	Bfsp2	beaded filament structural protein 2, phakinin	0,116	3,6E-03
MGI:1916133	Ahsa2	AHA1, activator of heat shock 90kDa protein ATPase homolog 2	0,115	1,4E-02
MGI:1919715	Zfp131	zinc finger protein 131	0,112	1,9E-02
MGI:1916523	Mettl7a1	methyltransferase like 7A1	0,110	1,9E-02
MGI:97314	Nefm	neurofilament, medium polypeptide	0,110	1,6E-02
MGI:1914167	Chordc1	cysteine and histidine-rich domain (CHORD)-containing, zinc-binding protein 1	0,109	2,0E-02
MGI:1917814	Fam213a	family with sequence similarity 213, member A	0,109	1,5E-02
MGI:1923691	Daam2	dishevelled associated activator of morphogenesis 2	0,109	1,4E-02
MGI:1914662	Soga3	SOGA family member 3	0,108	1,7E-02
MGI:2443958	A330023F24Rik	RIKEN cDNA A330023F24 gene	0,108	2,5E-02
MGI:1328308	Scrg1	scrapie responsive gene 1	0,107	2,4E-02
MGI:1309528	Ppp1r12a	protein phosphatase 1, regulatory (inhibitor) subunit 12A	0,107	2,6E-02
MGI:1916216	Ngdn	neuroguidin, EIF4E binding protein	0,106	2,7E-02
MGI:1922985	Pank1	pantothenate kinase 1	0,105	2,9E-02
MGI:1353450	Aldh1a1	aldehyde dehydrogenase family 1, subfamily A1	0,105	2,1E-02
MGI:2181202	Pfkfb3	6-phosphofructo-2-kinase/fructose-2,6-biphosphatase 3	0,105	2,5E-02
MGI:1890410	Acss2	acyl-CoA synthetase short-chain family member 2	0,104	2,4E-02
MGI:1928760	Ruvbl1	RuvB-like protein 1	0,104	2,9E-02
MGI:1921450	2810403A07Rik	RIKEN cDNA 2810403A07 gene	0,104	2,4E-02
MGI:1919103	Pdia6	protein disulfide isomerase associated 6	0,103	3,0E-02
MGI:97463	P4ha1	procollagen-proline, 2-oxoglutarate 4-dioxygenase (proline 4-hydroxylase), alpha 1 polypeptide	0,103	3,3E-02
MGI:1914538	Srek1ip1	splicing regulatory glutamine/lysine-rich protein 1interacting protein 1	0,102	3,4E-02
MGI:1914228	Luc7l	Luc7 homolog (S. cerevisiae)-like	0,101	2,6E-02
MGI:109130	Stip1	stress-induced phosphoprotein 1	0,101	2,4E-02
MGI:104680	Hspe1	heat shock protein 1 (chaperonin 10)	0,101	3,3E-02
MGI:1923429	Usp31	ubiquitin specific peptidase 31	0,100	2,4E-02

MGI:95834	Pdia3	protein disulfide isomerase associated 3	0,100	2,9E-02
MGI:1917581	Shprh	SNF2 histone linker PHD RING helicase	0,100	3,8E-02
MGI:2682254	Zbtb40	zinc finger and BTB domain containing 40	0,099	2,3E-02
MGI:1929658	Tbk1	TANK-binding kinase 1	0,099	3,7E-02
MGI:95739	Glul	glutamate-ammonia ligase (glutamine synthetase)	0,099	1,9E-02
MGI:104864	Pdia4	protein disulfide isomerase associated 4	0,099	4,0E-02
MGI:1926176	Gas2l1	growth arrest-specific 2 like 1	0,098	3,7E-02
MGI:2445356	Smox	spermine oxidase	0,098	3,8E-02
MGI:2443430	Dgki	diacylglycerol kinase, iota	0,097	4,0E-02
MGI:2136381	Hadhb	hydroxyacyl-Coenzyme A dehydrogenase/3-ketoacyl-Coenzyme A thiolase/enoyl-Coenzyme A hydratase (trifunctional protein), beta subunit	0,097	4,2E-02
MGI:2443012	Tns3	tensin 3	0,097	3,3E-02
MGI:1355331	Olig2	oligodendrocyte transcription factor 2	0,096	4,5E-02
MGI:1918764	Sfpq	splicing factor proline/glutamine rich (polypyrimidine tract binding protein associated)	0,096	3,9E-02
MGI:2429660	Tbc1d4	TBC1 domain family, member 4	0,095	4,5E-02
MGI:1931874	Dnajb1	DnaJ (Hsp40) homolog, subfamily B, member 1	0,095	4,6E-02
MGI:1914436	Rplp2	ribosomal protein, large P2	0,094	4,5E-02
MGI:1926462	Hnrnph3	heterogeneous nuclear ribonucleoprotein H3	0,094	4,8E-02
MGI:95640	Gapdh	glyceraldehyde-3-phosphate dehydrogenase	0,094	4,6E-02
MGI:1925496	Phf23	PHD finger protein 23	0,093	5,0E-02
MGI:1928739	Dnajb2	DnaJ (Hsp40) homolog, subfamily B, member 2	0,093	4,2E-02
MGI:1927406	Herpud1	homocysteine-inducible, endoplasmic reticulum stress-inducible, ubiquitin-like domain member 1	0,093	4,3E-02
MGI:98341	Snrnp70	small nuclear ribonucleoprotein 70 (U1)	0,092	2,5E-02
MGI:107380	Spag7	sperm associated antigen 7	0,092	4,9E-02
MGI:109522	Ugt8a	UDP galactosyltransferase 8A	0,091	4,2E-02
MGI:1923013	Dcaf17	DDB1 and CUL4 associated factor 17	0,090	4,6E-02
MGI:1891824	Acin1	apoptotic chromatin condensation inducer 1	0,090	4,6E-02
MGI:1353499	Baz1b	bromodomain adjacent to zinc finger domain, 1B	0,090	4,5E-02
MGI:96247	Hsp90ab1	heat shock protein 90 alpha (cytosolic), class B member 1	0,090	2,4E-02
MGI:1923686	Tufm	Tu translation elongation factor, mitochondrial	0,090	3,6E-02
MGI:106014	Nudc	nuclear distribution gene C homolog (Aspergillus)	0,088	4,7E-02
MGI:88025	Ank2	ankyrin 2, brain	0,088	3,4E-02
MGI:95524	Fgfr3	fibroblast growth factor receptor 3	0,082	4,9E-02
MGI:1922942	Nr2c2ap	nuclear receptor 2C2-associated protein	0,080	2,4E-02
MGI:99517	Hspa1b	heat shock protein 1B	0,078	3,0E-02
MGI:1921997	Ddit4	DNA-damage-inducible transcript 4	0,077	4,6E-02

MGI:1203481	Map3k19	mitogen-activated protein kinase kinase kinase 19	0,074	4,4E-02
MGI:3643804	Gm5499	predicted pseudogene 5499	0,067	4,7E-02
MGI:1859388	Dll4	delta-like 4 (Drosophila)	0,061	4,0E-02
MGI:2685450	Rtp1	receptor transporter protein 1	0,059	8,3E-03
MGI:1930153	Popdc3	popeye domain containing 3	0,058	2,6E-02
MGI:2685841	Mamdc4	MAM domain containing 4	0,058	2,7E-02
MGI:5504060	Gm26945	predicted gene, 26945	0,053	8,1E-03
MGI:109619	Neurog2	neurogenin 2	0,046	3,5E-02
MGI:3646759	Zfp947	zinc finger protein 947	0,032	2,3E-02
MGI:3704330	Gm10177	predicted gene 10177	0,029	1,8E-02
MGI:1915594	Tmem174	transmembrane protein 174	0,027	3,1E-02
MGI:96090	Asmt	acetylserotonin O-methyltransferase	-0,014	4,8E-02
MGI:96177	Hoxa5	homeobox A5	-0,020	2,1E-02
MGI:3649000	Gm8104	predicted gene 8104	-0,024	4,8E-02
MGI:2443002	A330032B11Rik	RIKEN cDNA A330032B11 gene	-0,026	4,8E-02
MGI:3045296	Efhb	EF hand domain family, member B	-0,026	2,5E-02
MGI:1923110	Tex26	testis expressed 26	-0,027	4,1E-02
MGI:1196234	Ccdc69	coiled-coil domain containing 69	-0,040	3,5E-02
MGI:3649668	Gm13544	predicted gene 13544	-0,040	1,5E-02
MGI:4439718	Gm16794	predicted gene, 16794	-0,051	4,5E-02
MGI:1346063	Ggt5	gamma-glutamyltransferase 5	-0,063	4,8E-02
MGI:95557	Flnc	filamin C, gamma	-0,069	4,8E-02
MGI:1855694	Isg15	ISG15 ubiquitin-like modifier	-0,069	4,6E-02
MGI:2441887	Gpr151	G protein-coupled receptor 151	-0,078	4,6E-02
MGI:1921330	Nmnat3	nicotinamide nucleotide adenylyltransferase 3	-0,078	3,8E-02
MGI:1915025	Rtp4	receptor transporter protein 4	-0,080	2,5E-02
MGI:1926263	Gbp3	guanylate binding protein 3	-0,083	9,7E-03
MGI:1913292	Sostdc1	sclerostin domain containing 1	-0,084	3,3E-02
MGI:1929063	Copz1	coatomer protein complex, subunit zeta 1	-0,084	4,9E-02
MGI:1888992	Msln	mesothelin	-0,088	1,6E-03
MGI:102643	Myh11	myosin, heavy polypeptide 11, smooth muscle	-0,089	1,2E-02
MGI:1914500	Ube2l6	ubiquitin-conjugating enzyme E2L 6	-0,089	4,7E-02
MGI:894315	Rap1b	RAS related protein 1b	-0,089	4,1E-02
MGI:1915288	Chid1	chitinase domain containing 1	-0,090	4,5E-02
MGI:1859682	Slc23a2	solute carrier family 23 (nucleobase transporters), member 2	-0,091	3,6E-02
MGI:97386	Ntsr1	neurotensin receptor 1	-0,091	4,8E-02
MGI:88501	Crip1	cysteine-rich protein 1 (intestinal)	-0,092	3,4E-02
MGI:104745	Sez6	seizure related gene 6	-0,093	2,8E-02
MGI:2148924	Clic1	chloride intracellular channel 1	-0,093	4,8E-02
MGI:2145316	Txndc5	thioredoxin domain containing 5	-0,094	3,0E-02
MGI:1344344	Dazap2	DAZ associated protein 2	-0,095	2,2E-02
MGI:95895	H2-Aa	histocompatibility 2, class II antigen A, alpha	-0,095	2,4E-02
MGI:1913625	Dhrs7	dehydrogenase/reductase (SDR family)	-0,095	4,8E-02

		member 7		
MGI:1353596	Tor2a	torsin family 2, member A	-0,095	4,6E-02
MGI:97742	Pomc	pro-opiomelanocortin-alpha	-0,095	5,3E-03
MGI:106214	Jrk	jerky	-0,095	4,2E-02
MGI:103156	Gabrg1	gamma-aminobutyric acid (GABA) A receptor, subunit gamma 1	-0,096	4,6E-02
MGI:98476	Tac2	tachykinin 2	-0,096	4,4E-02
MGI:97168	Msx1	msh homeobox 1	-0,097	1,9E-02
MGI:1277179	Pde9a	phosphodiesterase 9A	-0,097	4,4E-02
MGI:1914164	Vps28	Vacuolar protein sorting 28	-0,097	3,5E-02
MGI:2157946	Cacng5	calcium channel, voltage-dependent, gamma subunit 5	-0,098	4,2E-02
MGI:2386741	Slc30a6	solute carrier family 30 (zinc transporter), member 6	-0,098	4,1E-02
MGI:2685790	Garem	GRB2 associated, regulator of MAPK1	-0,098	3,8E-02
MGI:2146851	Tfb1m	transcription factor B1, mitochondrial	-0,099	2,1E-02
MGI:2138915	Myl9	myosin, light polypeptide 9, regulatory	-0,100	3,5E-02
MGI:102709	Cav1	caveolin 1, caveolae protein	-0,100	3,7E-02
MGI:3045495	Tmem205	transmembrane protein 205	-0,100	3,4E-02
MGI:103017	Fcgrt	Fc receptor, IgG, alpha chain transporter	-0,100	3,7E-02
MGI:1913951	Spryd4	SPRY domain containing 4	-0,101	3,3E-02
MGI:2136782	BC004004	cDNA sequence BC004004	-0,102	2,4E-02
MGI:2385957	Mfrp	membrane-type frizzled-related protein	-0,104	6,7E-03
MGI:1914081	Cab39l	calcium binding protein 39-like	-0,105	2,9E-02
MGI:1913391	Ifitm3	interferon induced transmembrane protein 3	-0,107	2,0E-02
MGI:1913301	0610012G03Rik	RIKEN cDNA 0610012G03 gene	-0,107	2,5E-02
MGI:3582583	Sprn	shadow of prion protein	-0,107	1,0E-02
MGI:95405	Ephx1	epoxide hydrolase 1, microsomal	-0,111	2,1E-02
MGI:1096574	Car4	carbonic anhydrase 4	-0,113	1,8E-02
MGI:1913974	Tab3	TGF-beta activated kinase 1/MAP3K7 binding protein 3	-0,114	1,7E-02
MGI:1915376	Fahd2a	fumarylacetoacetate hydrolase domain containing 2A	-0,115	1,7E-02
MGI:1860292	Ramp3	receptor (calcitonin) activity modifying protein 3	-0,115	1,6E-02
MGI:2147570	Marveld1	MARVEL (membrane-associating) domain containing 1	-0,117	1,1E-02
MGI:1341868	Rfc2	replication factor C (activator 1) 2	-0,118	1,2E-02
MGI:95904	H2-K1	histocompatibility 2, K1, K region	-0,119	1,3E-02
MGI:98822	Tfrc	transferrin receptor	-0,120	1,3E-02
MGI:1101771	Kl	klotho	-0,121	1,0E-02
MGI:1351317	Podxl	podocalyxin-like	-0,122	1,1E-02
MGI:894696	Serping1	serine (or cysteine) peptidase inhibitor, clade G, member 1	-0,126	6,0E-03
MGI:99554	Lgals3bp	lectin, galactoside-binding, soluble, 3 binding protein	-0,128	6,8E-03
MGI:88498	Crhr1	corticotropin releasing hormone receptor 1	-0,129	7,3E-03
MGI:98865	Ttr	transthyretin	-0,130	5,7E-03
MGI:1352480	Igfbp7	insulin-like growth factor binding protein 7	-0,130	6,5E-03

MGI:99261	Ptgds	prostaglandin D2 synthase (brain)	-0,131	1,3E-03
MGI:106012	Tagln	transgelin	-0,146	1,9E-03
MGI:106679	Zic2	zinc finger protein of the cerebellum 2	-0,152	1,6E-03
MGI:97290	Ndn	necdin	-0,165	1,6E-04
